# The role of cell location and spatial gradients in the evolutionary dynamics of colon and intestinal crypts

**DOI:** 10.1186/s13062-016-0141-6

**Published:** 2016-08-23

**Authors:** Leili Shahriyari, Natalia L. Komarova, Alexandra Jilkine

**Affiliations:** 1 Mathematical Biosciences Institute, The Ohio State University, 1735 Neil Ave, Columbus, 43210 USA; 2 Department of Mathematics, University of California Irvine, 340 Rowland Hall, Irvine, 92697 USA; 3 Department of Applied and Computational Mathematics and Statistics, University of Notre Dame, 153 Hurley Hall, Notre Dame, 46556 USA

**Keywords:** Intestinal crypt, Stochastic model, Mutation accumulation, Symmetric division, Carcinogenesis, Two hit mutant, Optimization, BrdU labeling

## Abstract

**Background:**

Colon and intestinal crypts serve as an important model system for adult stem cell proliferation and differentiation. We develop a spatial stochastic model to study the rate of somatic evolution in a normal crypt, focusing on the production of two-hit mutants that inactivate a tumor suppressor gene. We investigate the effect of cell division pattern along the crypt on mutant production, assuming that the division rate of each cell depends on its location.

**Results:**

We find that higher probability of division at the bottom of the crypt, where the stem cells are located, leads to a higher rate of double-hit mutant production. The optimal case for delaying mutations occurs when most of the cell divisions happen at the top of the crypt. We further consider an optimization problem where the “evolutionary” penalty for double-hit mutant generation is complemented with a “functional” penalty that assures that fully differentiated cells at the top of the crypt cannot divide.

**Conclusion:**

The trade-off between the two types of objectives leads to the selection of an intermediate division pattern, where the cells in the middle of the crypt divide with the highest rate. This matches the pattern of cell divisions obtained experimentally in murine crypts.

**Reviewers:**

This article was reviewed by David Axelrod (nominated by an Editorial Board member, Marek Kimmel), Yang Kuang and Anna Marciniak-Czochra. For the full reviews, please go to the Reviewers’ comments section.

**Electronic supplementary material:**

The online version of this article (doi:10.1186/s13062-016-0141-6) contains supplementary material, which is available to authorized users.

## Background

In adult tissues, a balance must exist between stem cell proliferation and the production of differentiated offspring. To maintain homeostasis between cell types, on average half of all stem cell offspring must differentiate, and the remaining half must maintain their stem cell identity. The concept of a stem cell that always divides asymmetrically, producing one stem and one differentiated cell has recently been challenged [1, 2], and in many tissues some fraction of stem cell divisions have been shown to be symmetric [3]. The exact percentage of symmetric divisions depends on the tissue (estimated as 16 % in epidermis [4], 50 % for intestine [5], 100 % in germline cells [6]). In this process, stem cells are now thought to be routinely lost and replaced in a stochastic manner, suggesting that a stochastic model is necessary to understand stem cell evolutionary dynamics [7]. The strongest evidence for the stochastic nature of the division process has been demonstrated for intestinal crypts, where neutral competition between cells has been shown to lead to monoclonal conversion, i.e., ultimately all the cells in a crypt become descendants of a single stem cell [2].

Dividing stem cell populations face multiple performance objectives, such as steady state robustness (low sensitivity to parameter variation) [8, 9], minimizing fluctuations in the population size (low variance) [10, 11], rapid regeneration of population following injury [12], and delaying the onset of cancer [13, 14]. Rapidly dividing tissues, such as epithelial cells of intestinal crypts, are particularly sensitive to somatic mutation accumulation, which can lead to cancer. It is not clear which division strategies stem cells should follow to minimize the accumulation of mutations. Furthermore, for intestinal crypts there are reports that an age-dependent transition occurs from mainly asymmetric divisions to largely symmetric divisions [15], and opposing reports that a transition occurs from symmetric to asymmetric divisions [16]. The potential tradeoffs between symmetric and asymmetric stem cell divisions in tissues have previously been considered by many modelers using ODE models [17], stochastic space-free models [18–22], and in a spatial setting [23]. Several of these models consider the time to appearance of *k* consecutive mutations in a population of cells, and ask how mutation accumulation can be minimized. One common type of model used to study time to cancer initiation is a multi-type Moran process to model a constant cell population of size *N*, where the mutations can be disadvantageous, neutral, or advantageous. This waiting time depends on both population size *N* and mutation rate *u*. A modified Moran model shows that mutations that increase the probability of asymmetric division, lowering the likelihood of a symmetric division, can lead to rapid mutant stem cell expansion [18]. Shahriyari and Komarova [20] found that symmetrically dividing cells may delay double hit mutations compared to an equivalent system with asymmetric mutations. The rationale is that symmetric division producing two progenitors flushes out mutations in the stem cell lineage, if progenitor turnover is fast [19, 20, 22, 24].

**Fig. 1:**
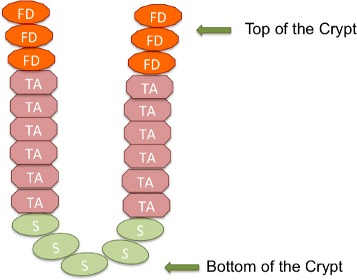
Schematic of an intestinal crypt. The intestinal crypt consists of several different types of cells. At the base of the crypt, stem cells are found within the stem-cell niche, with the niche formed by the stem cells themselves and mesenchymal cells that surround the crypt base. At the *top* of the crypt, there are fully differentiated cells, and between the *bottom* and the top of the crypt there are transit amplifying cells

Considering the spatial arrangement of stem cells further complicates the division process. In a space-free model, a newly arisen mutant competes with the entire population when reproducing. However, if a mutant only competes with cells in its local environment, that changes the time to acquisition of multiple mutations [25, 26]. The spatial geometry of the stem cell niche is best understood for colon/intestinal crypts [27, 28]. Colonic crypts have a similar spatial organization of cell types (stem cells, proliferating transient amplifying cells, and differentiated cells) as intestinal crypts. We focus on both of those tissue types in this paper. The stem cells are found at the bottom of a crypt (see Fig. 1). Cell division and placing of progeny cells leads to shuffling of cell positions in the crypt. Daughter cells that stay in the same position remain a stem cell, while those that move up the crypt, exit the stem cell compartment and become transit amplifying (TA) cells [5]. TA cells undergo approximately four to five rounds of division every 12 hrs and differentiate into more specialized cell types, such as enterocytes, goblet cells and enteroendocrine cells, which continue to move up [29]. This upward cell movement is similar in intestinal and colonic crypts. However, at the top of the colonic crypt, the fully differentiated cells are shed into the lumen and transported away, whereas cells at the top of an intestinal crypt move up to a villus and are removed at the top of the villus. The cells at the top are replaced by a combination of active cell migration and passive motion due to divisions [30, 31].

A first step toward a spatial model includes two stem cell compartments with different properties, determined by their proximity to the base of the niche. Ritsma et al. [5] found two distinct groups of stem cells in intestinal crypts: the ‘border cells’ located in the upper part of the niche at the interface with TA cells, and ‘central cells’ located at the crypt base, with different proliferative potentials. Motivated by this finding, a bi-compartmental niche model with two types of stem cells was considered in [32]. Again, in this simplified version of a spatial model symmetrically dividing cells were found to generate double-hit mutants at a lower rate than asymmetrically dividing cells.

There have been multiple computational models of the colonic and intestinal crypts that take spatial location into account [33–38]. Bravo and Axelrod [37] and Kagawa et al. [38] developed agent-based models that included stem cells, proliferating cells, and differentiated cells. These models were calibrated by experimental measurements of cells in biopsies of normal human colon crypts, and demonstrated realistic quasi-stationary crypt dynamics. Similar multiscale models recapitulate experimentally observed steady state cell distribution in intestinal crypts [33] and examine hypotheses for how cell differentiation and proliferation is regulated through Wnt and Notch signals [36]. The crypt geometry has a significant impact on the time it takes for a crypt to reach mono-clonality [34]. When the spatial location of the initial mutation was varied, mutations more than one or two cell layers away from the base of the crypt were found unlikely to become a dominant clone, and the ability of a mutant clone to take over a crypt is extremely sensitive to the position at which the mutation occurs [35]. However, these virtual crypt models do not provide any analytical results, and new simulations have to be performed if a parameter is altered.

A linear process version of a Moran model, where analytical results can be obtained, has also been considered [23, 25, 39]. Proliferation kinetics with proliferative activity concentrated at the bottom of the crypt were found to have a greater ability of delaying the rate of mutation accumulation in a linear process model compared to proliferation curves near the top of the crypt [23]. However, in that model no distinction was made between symmetric and asymmetric division, with a daughter cell having equal probability of occupying position *i* or *i*+1. We are not aware of a spatial model of a crypt with both symmetric and asymmetric divisions in the mathematical literature.

In this article, we investigate the role of spatial structure on two-hit mutant production in a stochastic model of the colon/intestinal crypt, in the context of symmetric and asymmetric divisions patterns. We consider a two-mutant cell to correspond to a cell where a tumor suppressor gene has been inactivated, such as an APC ^−/−^ cell that is able to break out of homeostatic control and resist shedding from the crypt [40]. It is thought that those cells will be retained in the crypt and can lead to formation of adenomas. Note also that a simple calculation shows that a two hit cell is likely to occur in the TA cell compartment [41]. However, that calculation did not include spatial dependence of division likelihood within a crypt. In this paper, we assume that cells at different locations have different probabilities of division. We vary the probability of division function to investigate whether location of dividing cells has an effect on double hit mutant generation. We consider cases where cells at the bottom of the crypt have higher probability of division than cells at the top of the crypt, and the opposite scenario where cells near the top of the crypt divide more frequently, as well as intermediate cases. We calculate the probability of a two-hit mutant appearing for an empirically determined proliferation curve in murine intestinal crypts. We consider two types of optimization problem. In one, the objective is to minimize the rate of double-hit mutant production. In the second one, this “evolutionary” objective is counterbalanced with a “functional” objective to make sure that more differentiated cells divide less frequently. The resulting trade-off may explain the observed patterns of cell divisions in colonic crypts.

## Methods

### Model set-up

**Fig. 2:**
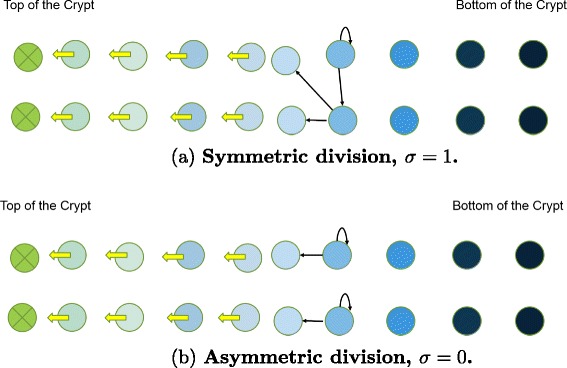
Spatial model of the crypt with both symmetric and asymmetric divisions. Here, *yellow arrows* show the direction of cell migration, and *black arrows* show the divisions. The tail of each *black arrow* indicates the location of the dividing cell, and the head of the *arrow* shows the location of the offspring. At each time step, two cells at the *top* of crypt die, then two cells at position *x* divide. Cells are chosen for division based on the probability division function *p*_*div*_(*i*). Note that only one position *i* is chosen to undergo division. All the cells at that position undergo division. When a cell divides, it pushes cells above toward the *top* of the crypt. Here, colors indicate degree of differentiation, with darker colors indicating stem cells. The differentiation level of the cells only affects their probability of undergoing division *p*_*div*_(*i*). A symmetric division produces two cells of equal differentiation level as their mother cell. The symmetric pattern couples a differentiation event (daughter cells are placed upstream from the division location) and proliferation event (daughter cells are placed at the same level as the dividing cell). In the asymmetric division pattern, one of daughter cells stays at the same location and the other one is placed upstream

Here, we develop a spatial stochastic model of the crypt, that is a generalization of the Moran process [42, 43]. The total cell population remains constant, in line with what is observed in normal intestinal crypts, whose size is remarkably constant [29]. The test-tube shaped crypt (see Fig. 1) can be approximated by a cylinder and ‘unrolled’ onto a flat planar domain with periodic left- and right-hand boundaries. We are only concerned with differences between cells in the z-direction, hence model the crypt with “linear process” quasi-1D array of cells from [39]. However, to accommodate both symmetric and asymmetric divisions, our model includes two rows of *n* cells (Fig. 2). This allows a symmetrically dividing cell to place two progeny at the same distance from the crypt bottom.

In our model, cell death is being modeled as it occurs in colon crypts. At each updating time step, the two cells at the top of the crypt die and then two cells at position *x* in the crypt are chosen to divide, according to the division gradient probability function *p*_*div*_(*x*). The cells upstream from the newly divided cells will move up toward the top of the crypt in order to fill out the empty space and open a space for the new daughter cells. We denote the index of columns by *x*, so that cells at the position *x*=0 corresponds to the cells at the top of the crypt and *x*=*n*−1, the bottom of the crypt. We assume that cell death always happens in the position *x*=0, and nowhere else. The effect of relaxing this assumption will be considered at the end of the Results section. At each updating step, one position *x*=*m*, 0<*m*<*n* is randomly chosen for division based on the division probability function. When a cell at position *x*=*m* divides, then each cell at the position *x* with 0<*x*<*m* migrates to location *x*−1, and other cells at the positions *x*>*m* do not change. When a wild type cell divides, with probability *u*_1_ a mutation happens and one of daughter cells becomes a 1-hit mutant. The first mutation is assumed to be neutral, i.e. does not affect *p*_*div*_(*x*). With probability *u*_2_ one of a 1-hit mutant’s daughter cells becomes a 2-hit mutant.

The relative proportion of symmetric divisions can vary and is denoted by *σ*, where *σ*=1 means that all divisions are symmetrical, and *σ*=0 means that all cells divide asymmetrically. The proliferation and differentiation events are coupled so that the total cell population remains constant (Fig. 2). When an asymmetric division occurs at position *x*=*i*, one of the progeny replaces the cell at *x*=*i*, and the other progeny is placed at *x*=*i*−1. When a symmetric division occurs at position *i*, both progeny from one row are placed at position *m* (i.e., proliferation event where the progeny remain at the same position as the mother cell), and the progeny from the neighbor row are placed at position *m*−1 (differentiation event, where the progeny move up the crypt). Hence, symmetric divisions produce two cells of the same type, either at the same differentiation level as the mother (proliferation event), or a more differentiated type (differentiation event). Asymmetric divisions produce one daughter cell of the same type, and one daughter cell that is more differentiated.

### Division gradient probability functions

**Fig. 3:**
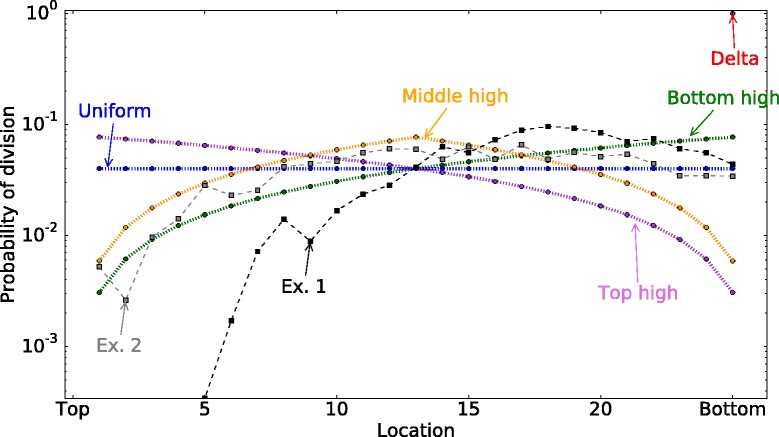
Division probability distribution functions. Each cell depending on its location has a different probability of division *p*_*div*_(*i*). In this figure, the total number of cells in a row is 26. The location *x*=0 corresponds to the *top* of the crypt and *x*=25 corresponds to the *bottom* of the crypt. Note that in this model cells at *x*=0, i.e. the *top* of the crypt, do not divide. We consider five different theoretical division probability functions and two experimentally measured functions Ex. 1 and Ex. 2. The latter two functions derive from the positional BrdU label index in the murine intestinal crypt from [33], obtained 2h and 24h after labelling, respectively
- **Top high**. Cells at the top of the crypt are dividing more than the cells at the bottom of the crypt, $p_{div}(x)=-\frac {2(x-n)}{n(n-1)}$ (decreasing function of x).
- **Bottom high**. Cells at the bottom of the crypt are dividing more than the cells at the top of the crypt, $p_{div}(x)=\frac {2x}{n(n-1)}$ (increasing function of x).
- **Delta**. At each time step, division happens only on the last column *x*=*n*−1, and other cells migrate toward the top of the crypt. *p*_*div*_(*x*)=*δ*(*x,n*−1).
- **Middle high**. The cells at the middle of the crypt are dividing more than the cells at the bottom and the top of the crypt, $p_{div}(x)=2\frac {1 - | 1- 2x/n |}{n}$.
- **Experimentally measured proliferation curves**. The experimental curves from [33] showing proliferation in murine intestinal crypts are shown in Fig. 3 as Experimental Curve 1 (obtained 2 hrs after labeling) and Experimental Curve 2 (24 hrs after labeling). See Parameter estimations section for details. A qualitatively similar distribution is seen for the human colon crypt [44].

In this model, cell are randomly chosen to divide depending on their location. The division probability of the cell at the position *x* is given by the probability distribution function *p*_*div*_(*x*), $\sum _{x=1}^{n-1} p_{div}(x)=1$. We compare the probability of two-hit mutant production for different *p*_*div*_(*x*) functions (Fig. 3):

- **Uniform**. All cells have the same probability to divide, i.e $ p_{div}(x)=\frac {1}{n-1}$

### The optimization problem

In this paper we consider two types of an optimization problem that can model selection pressures acting upon the colon crypt architecture. In the first problem, the only objective is to minimize the probability of a two-hit mutant generation by time *T*. We refer to this objective function as *F*^*evol*^:

$$F^{evol}=P_{2 hit}(T). $$

**Fig. 4:**
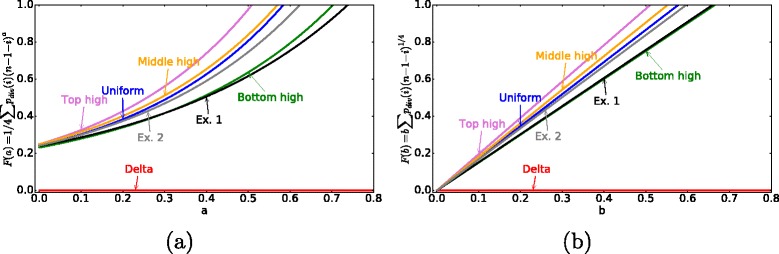
The penalty function $F^{func}=b \sum _{i=0}^{n-1} p_{div}(i) (n-1-i)^{a}$. In (**a**) *F* ^*func*^ is plotted a function of variable *a* with $b=\frac {1}{4}$. In (**b**) *F* ^*func*^ as a function of variable *b* with $a=\frac {1}{4}$. Note that 0<*F* ^*func*^<1

In the second optimization problem, we also include functional considerations in the model, by incorporating the effects of the proliferation ceiling. It is thought that a spatial gradient of extracellular Wnt along the crypt axis determines the position-dependent rates of cell differentiation [45]. The highest levels of Wnt signalling are observed in cells located at the crypt bottom decreasing gradually along the crypt’s z-axis. To take into account the fact that cells higher up in the crypt are more differentiated, and terminally differentiated cells cannot divide, a proliferation ceiling based on an external Wnt gradient is often imposed in computational models of the crypt [33, 35, 37, 46]. We consider the effect of cell differentiation in the crypt indirectly. As fully differentiated cells do not divide, let *F*^*func*^ be a “penalty” function for cells that divide at the top of the crypt (see Fig. 4). We take

$$ F^{func}=b \sum_{i} p_{div}(i) (n-1-i)^{a}, $$

where *a* and *b* are fixed constants. Note that the delta division pattern has no associated penalty, since all divisions happen at the bottom of the crypt. By varying *a* and *b*, we can obtain different values of *F*^*func*^ for a given division pattern *p*_*div*_(*i*), with the general property that proliferation curves with more divisions near the top of the crypt are penalized in Scenario 2. The second optimization setup aims to minimize the sum of the evolutionary and functional components,

$$F^{evol}+F^{func}. $$

Note that parameter *b* simply measures the relative weight of the “functional” penalty with respect to the “evolutionary” penalty. Parameter *a* is varied between 0 and 1. The value *a*=1 corresponds to the strongest dependence of the functionality on the cells’ positions along the crypt, and to *F*^*func*^ being proportional to the mean position of cell divisions in the crypt. The value *a*=0 corresponds to the absence of discrimination among the cell positions and the absence of the proliferation ceiling.

### Parameter estimates

The parameters used are summarized in Table 1. A rich source of data on the tissue renewal cycle in the colon/intestine are experimental studies of the mouse intestine. Adult murine crypts contain about 250-300 cells, with 5-7 actively dividing stem cells [47, 48]. In the murine small intestine, it takes 2-3 days for a cell to migrate up from the base of the crypt to the top, where it will be removed [49]. All crypt cells, excluding stem cells and Paneth cells in the niche at the bottom of the crypt, will be renewed over this period. The stem cells divide once a day [50]. TA cells undergo approximately four to five rounds of division approximately every 12 hrs [29].

**Table 1:** Model parameters for colon/intestinal crypts

Parameter	Estimated value	Ref.	Simulations
*n*: height of human colon crypt (number of cells in one column of crypt)	80	[53]	varied (2–80)
*n*: height of murine intestinal crypt (number of cells in one column of crypt)	25	[33]	
*σ*: probability of symmetric division	16 *%* in epidermis,	[4]	varied (0–1)
	50 *%* for intestine, 100 *%* in germline cells	[5, 6]	
*u*: mutation rate	10^−7^ in normal cells to 10^−2^ in the case of chromosomal instability	[68]	*u*_1_ varied from 0.0005 to 0.01
			*u*_2_ varied from 0.0001 to 0.02

Human crypt stem cell studies are limited because of the inability to use the cell fate mapping experimental techniques of model systems. However, there are also studies where parameters for human colon crypts have been inferred [51, 52]. Each crypt contains around 2000 cells, with about 40 cells in circumference and 80 cells in height [53]. It has been estimated that there are 5–6 actively dividing stem cells in the human colon crypt [52]. The stem cells divide once every 2–3 days [44]. The organization is similar to murine crypts, but the crypts are 2–4 times longer [54]. The intestinal stem cell in humans is estimated to divide as many as 5000 times during a lifetime [29].

There are some empirical data on cell proliferation data along the crypt axis obtained by measuring the fraction of BrdU+ cells in human colons following injection as a measure of average division rate [53]. (BrdU is a synthetic nucleotide (analogue of thymidine) that is usually applied by adding it to the drinking water of animals and/or by injection. Fluorescently marked antibodies that attach to BrdU are used to detect cells that are BrdU+. When BrdU+ cells divide without BrdU present, their label is diluted. Tracking the proportion of BrdU+ cells during both the uptake and loss period provides a mechanism to measure the turnover kinetics of a given population of cells.) The measured labeling curve from [53] shows that most mitotic activity is occurring in the lower part of a colon crypt. We also use published data from similar experiments on murine crypts (Fig. 3), where positional BrdU label index was obtained two and 24 hours after injection with BrdU [33]. Both murine and human crypts show a very similar pattern of divisions, with most divisions occurring in the lower part of the crypt, but not at the very bottom, where the stem cells are located. Reports that TA cells are dividing every 12 hours compared to every 24 hours for stem cells [29] also suggest that divisions are more frequent some distance away from the bottom of the crypt.

Mutation rate estimates (for inactivating mutations) per cell division per gene range from about 10^−7^ in normal cells to 10^−2^ in the case of chromosomal instability. (Note that the rate of epigenetic change has been estimated to be orders of magnitude higher than that of the normal genetic change and also plays a role in cancer initiation [51, 55].) We consider two mutations necessary for inactivation of a tumor suppressor gene such as APC, the most common mutation (80 % of patients) that is found in colorectal cancers [56]. In this paper we vary the mutation parameters *u*_1_ and *u*_2_. In particular, we consider the case where *u*_1_=*u*_2_ (that is, the first and the second hit are acquired at the same rate), and also consider the scenario where *u*_2_>*u*_1_, that is, the mutation rate *u*_2_ to get 2nd hit (APC ^+/−^ to APC ^−/−^) is higher than the mutation rate *u*_1_ to get the 1st hit (APC ^+/+^ to APC ^+/−^). This is because in the presence of chromosomal instability, the 2nd hit may occur due chromosome deletion, duplication, or gene conversion rather than a 2nd point mutation, resulting in a higher rate [57].

It has previously been proposed that intestinal stem cells use an *immortal strand mechanism* in order to minimize the accumulation of mutations in their genomes [58]. Recent evidence suggests that there is no immortal strand stem cell segregation in intestinal stem cells both through direct and indirect measurements [59–61]. Mutation rates found in stem cells are comparable to those expected without protection mechanism [62], so this is the assumption we make.

### Numerical simulations

Time is measured in cell divisions. At each time step, the cells at the location *x*=0 die. Location *x* for cell division is chosen according to the probability function *p*_*div*_. With probability *σ*, cells follow the symmetric division pattern, and with probability 1−*σ*, they follow the asymmetric division pattern. If a wild type cell divides then with probability *u*_1_, one of its daughter cells is mutated. If a 1-hit mutant divides, then with probability *u*_2_ one of its daughter cells becomes a 2-hit mutant. Note that if a mutation happens, only one of the daughter cells gains it, and both daughter cells have the same probability of mutation. Cells between division location *x* and the top of the crypt (location 0) are pushed to the top of the crypt. Hence, cells at location *m*, where 0<*m*<*x*, migrate to location *m*−1.

The majority of the simulations were run using the “linear process” geometry with two rows of *n* cells. However, we also consider even multiples of rows of *n* cells, with each pair of rows operating independently. In that case we simulated *k* two-rows of cells, where each row has *n* cells (such that the total number of cells in this simulation is *k*×(2×*n*), representing the cylindrical geometry of the crypt). At each updating time, one of *k* two-rows is chosen uniformly for two deaths and two divisions based on the proposed two-row model. For simplification, each two-row operates independently from the other one, so no periodical condition is imposed. Similar results were obtained and are shown in the figures.

#### The probability of two-hit mutant existence in at least one location $P_{2hit}^{gen}(T)$

We obtained the probability of double-hit mutant production in at least one location *x*>0 by time, *T*, denoted here by $P_{2hit}^{gen}$, where superscript *gen* refers to “generation”. We did not include location *x*=0 (the top of the crypt), because we assumed that at the next updating step cells at location 0 would undergo apoptosis. In order to calculate this probability, we stopped the repeat of updating steps when one of the following events happened: the first 2-hit mutant appeared in any location *x*>0 or the maximum time *T* was reached. We repeated this process 1000 times, and then calculated the proportion of the runs where a two-hit mutant was generated. This procedure was repeated 10 times, to calculate the mean and standard deviation.

**Fig. 5:**
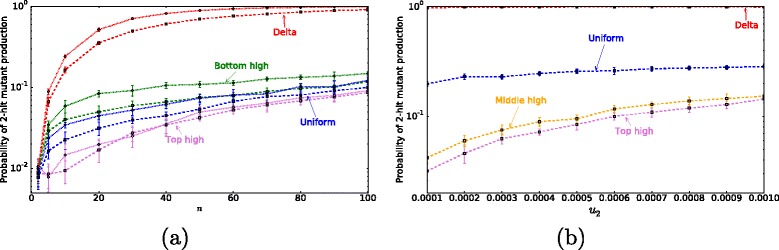
Probability of 2-hit mutant generation $P_{2 hit}^{gen}(T)$ at least in one location. Circles and squares are respectively results of the simulations for asymmetric (*circles, dotted lines*) and symmetric (*squares, dashed lines*) division for each division probability function. **a** $P_{2 hit}^{gen}(T)$ as the total number of cells in the crypt *n* is varied. Other parameters are *T*=100*n*, *u*_1_=0.0005, *u*_2_=0.002. **b** $P_{2 hit}^{gen}(T)$ as the second hit mutation rate *u*_2_ is varied. Other parameters are *t*=1000*n*, *u*_1_=0.0005, *σ*=0.5, and *n*=26

The total number of cells *n*, and the mutation rate *u*_2_ are varied in Fig. 5. The simulations were stopped as soon as the first copy of a double-hit mutant was produced, and the probability of mutant generation, $P_{2hit}^{gen}(T)$, was calculated. This is equivalent to the assumption that the mutant is significantly fitter than the rest of the cell population and a single product event will result in mutant invasion.

Although cells with inactivated APC may resist apoptosis and remain in the crypt [40], we assumed that a double-hit mutant at *x*=0 will be washed out of the crypt at the next time step. In this case, the presence of 2-hit mutants at *x*=0 could affect the dynamics after the mutant is generated. We consider the case where mutants can also be generated at *x*=0 and find the results for $P_{2hit}^{gen}$ to be qualitatively unchanged (see Additional file 1: Figure S1.)

#### The probability of two-hit mutant existence at each location $P_{2hit}^{exist}(x,T)$

**Fig. 6:**
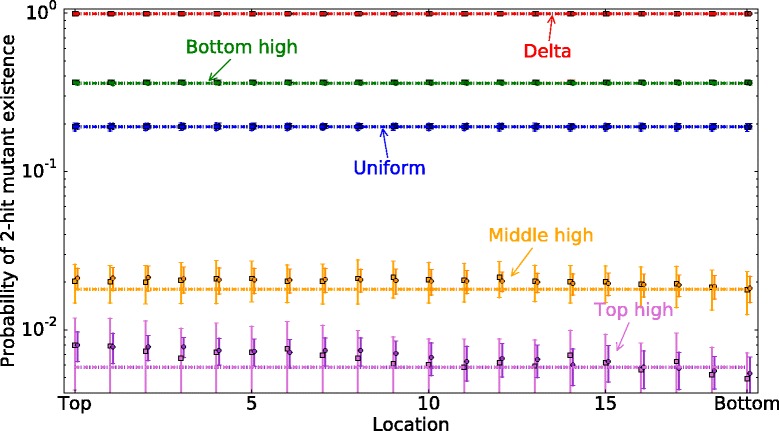
Probability of 2-hit mutant existence at each location, $P_{2hit}^{exist}(x,T)$. *Circles* and *squares* are respectively results of the simulations for asymmetric and symmetric divisions. *Dashed lines* are analytical results for symmetric divisions, and *dotted lines* are analytical results for asymmetric divisions (see equation (22) in Additional file 1). Note that *dashed lines* and *dotted lines* almost coincide for each division probability function. Parameters are *T*=10000, *u*_1_=0.001, *u*_2_=0.02, *n*=20

We also calculated the probability of two-hit mutant existence at each location, $P_{2hit}^{exist}(x,T)$. In Fig. 6, we plot the probability of 2-hit mutant existence at different locations, for different division probability distribution functions. An analytical treatment of this quantity is found in the Additional file 1.

To calculate $P_{2hit}^{exist}(x,T)$, we ran the updates until the maximum time *T* is reached. Then for each location, we checked the existence of a two-hit mutant. This procedure was repeated 1000 times. For each location, we calculated the proportion of the runs that resulted in a double-hit mutant. Then we repeated this process 10 times, to obtain the mean and standard deviation of these values. Note that the quantity $P_{2hit}^{exist}(x,T)$ (probability of mutant existence in a given location at time *T*) is quite different from the quantity $P_{2hit}^{gen}(T)$ (probability of mutant generation by time *T*, in any location). The difference is two-fold. First, $P_{2hit}^{exist}(x,T)$ is “local” (depends on *x*), and $P_{2hit}^{gen}(T)$ is “global” (generation in *any* location). Second, when calculating the quantity $P_{2hit}^{exist}(x,T)$, we do not suppress the dynamics of double-hit mutants. In other words, we do not stop updating steps when the first 2-hit mutant is generated, but instead run the program for a fixed number of time-steps, *T*. Thus, 2-hit mutants could be generated and washed out from the crypt before reaching time *T*, or they can be generated, migrate, divide, and produce clones. Note that the dynamics of two-hit mutants in this case is assumed to be neutral, that is, the division and death rates of double-hit mutants are identical to those of normal cells and one-hit mutants.

Is it reasonable to assume that the two-hit mutation will be neutral? Recent work [48] empirically obtained the probability *P*_*R*_ that a mutant stem cell replaces its neighbor for various common mutations in colon cancer. Based on their experiment the fitness of one-hit mutant Apc ^+/−^ is 1.6, while the fitness of two-hit mutant Apc ^−/−^ is 3.8. Note, however, that our model is not specific to Apc. Other genes that are frequently mutated in colon cancer, have their fitness dependent on the environment. For example, the fitness of P53 two-hit mutants is 0.9 in the normal colon, while, in the inflammatory environment, the fitness of P53 two-hit mutant is 1.4. Hence, the assumption that two-hit mutant is neutral, in this case, is reasonable.

## Results

**Probability of 2-hit mutant generation is minimized when most cell divisions occur at the top of the crypt**

**Fig. 7:**
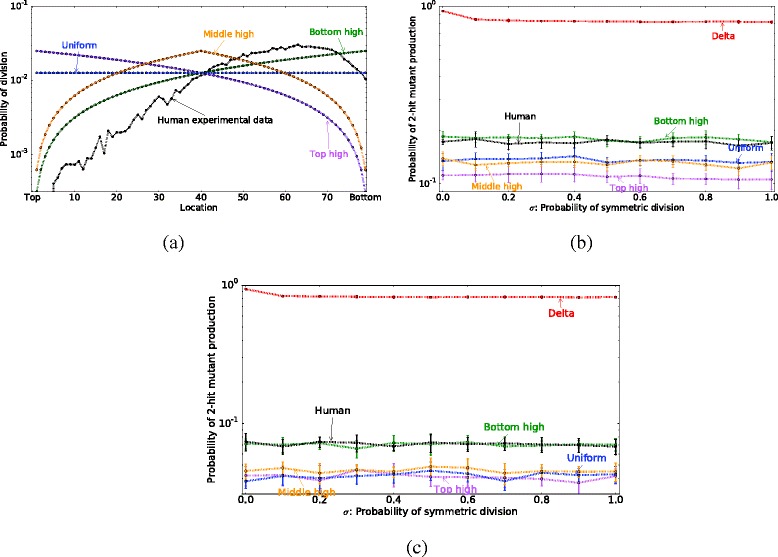
Simulations for the human colon crypt. **a** Division probability distribution functions for human colon crypt. Each cell depending on its location has a different probability of division *p*_*div*_(*i*). Here, the total number of cells in a row is 80. The location *x*=0 corresponds to the top of the crypt and *x*=79 corresponds to the *bottom* of the crypt. We consider five different theoretical division probability functions and one experimentally measured function. The human experimental function obtained from the positional BrdU label index in the human colon crypt from [44]. **b** In this plot, we assume only cells at the location *x*=0 die. **c** Here, 10 *%* of the time a uniform random cell death happens within the crypt. At each updating time step with probability 0.1 a location *x*, where 0<*x*<*n*−1, is chosen uniformly randomly for cells death. When two cells at the location *x* die, then a random cell located lower than the dead cells divide. This implies, the death cannot happen at very bottom of the crypt, i.e. the location *x*=*n*−1, because there is no cells located lower than this location to divide. The location of the division is chosen based on the normalized division probability function. For example, if the cells at the location *x*=*a* die, then the location of the divisions is chosen based on the probability function $\frac {p_{div}(x)}{S}$, where *a*<*x*<*n* and $S=\sum _{i=a}^{n-1} p_{div}(x)$. In both plots (**b**) and (**c**), we obtain the probability of 2-hit mutant generation $P_{2 hit}^{gen}(T)$ at least in one location as the probability of symmetric division *σ* is varied. Other parameters are *t*=4000, *u*_1_=0.001, *u*_2_=0.01, *n*=80. In these simulations, we model an entire human colon crypt (i.e. 12 two-rows), so the total number of cells is 1920

We have examined the probability of 2-hit mutant generation under different division probability distribution functions (Fig. 3). It it found that “bottom-high” division probability distributions correspond to lower probabilities of 2-hit mutant generation compared with “top-high”. Figure 5a shows the probability of 2-hit mutant generation by time *T* as a function of *n*. Different lines correspond to different division probability distribution functions, and both purely symmetric (*σ*=1) and purely asymmetric (*σ*=0) divisions patterns are shown. Figure 7b plots the probability of mutant production as a function of *σ*, the probability of symmetric cell divisions. Figure 5b represents the probability of 2-hit mutant generation as a function of the 2nd mutation rate. The division probability distribution given by the delta function always produces the highest probability of mutant generation, which is followed by the “bottom-high” probability distribution, which corresponds to predominantly bottom cells dividing in the system. The lowest probability of mutant generation corresponds to the “top-high” probability distribution.

A similar result is obtained when we look at the probability of mutant existence at different locations in Fig. 6. Again, the probability of mutant existence is maximized for the delta-function division probability distribution (followed by the “bottom-high” distribution), and it is minimized for the “top-high” function. It is interesting that the probability to find a two-hit mutant in this setting is independent of the location, *x*. As expected, the probability of one-hit mutant production mirrors the probability of division (Additional file 1: Figure S2). The probability of finding a 2-hit mutant shows a different trend. The reason for this is the neutral drift dynamics of one-hit and double-hit mutants that takes place in the system and evens out the probabilities of finding a 2-hit mutants at different locations. We see that for large *t*, *P*_2_(*x,t*)≈*P*_2_(*x*+1,*t*). This is confirmed by analytical results (see Additional file 1).

Figures 5 and 6 show two opposite assumptions on the double-hit mutant behavior. Figure 6 plots the probability of mutant existence, $P_{2hit}^{exist}$, where the double-hit mutants were assumed to be neutral, that is, possess exactly the same division and death dynamics as the rest of the cells in the population. Both assumptions lead to the same conclusion: to minimize the double-hit mutant production, cell divisions should happen according to the top-high pattern, that is, cells near the top of the crypt should divide the most.

**Fig. 8:**
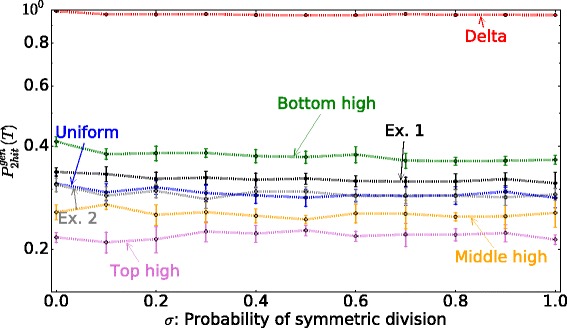
The optimal division pattern when the evolutionary optimization function is $F^{evol}=P_{2hit}^{gen}(T)$ for a murine intestinal crypt. The probability of 2-hit mutant generation is plotted for different probabilities of symmetric division, *σ*, for different division probability distribution functions. We connect the results of simulations by *dotted lines* for each division probability function. The parameters are *T*=2000, *u*_1_=0.005, *u*_2_=0.005, *n*=26. The total number of cells in this simulation is 312 (i.e. 6 two-rows), which is roughly the number of cells in a murine crypt. The lines Exp. 1 and Exp. 2 correspond to the division probability function that we obtained from experimental data in [33]

We note that the results reported here do not depend on the assumptions with regards to the relative mutation rate magnitude. For Figs. 5a and 7b we assummed that a second mutation is more likely once a first mutation has occurred (i.e., *u*_1_<*u*_2_), as discussed in Parameter Estimates section. A similar result is obtained for *u*_1_=*u*_2_ and *u*_1_>*u*_2_. In Fig. 5b we vary the value of the 2nd mutation *u*_2_ over a range of values and find that the top high division pattern always results in a lower probability of 2-hit mutant production. In summary, if the only evolutionary objective is to minimize the probability of double-hit mutant production (taking $F^{evol}=P_{hit}^{gen}(T)$), the best strategy corresponds to all divisions happening at the very top of the crypt, regardless of cell number *n*, proportion of symmetric divisions *σ*, and mutation rate *u*_2_. Using this criterion, the bottom high division pattern outperforms the experimentally observed division pattern (Fig. 8). Since the probability of double hit mutations are independent of location, the crypt dynamics are not the most efficient as they could be in minimizing double hit neutral mutations.

**Including the proliferation ceiling makes the experimentally observed division pattern most optimal**

**Fig. 9:**
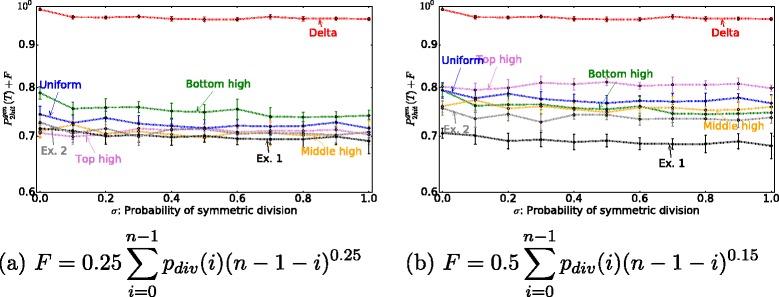
The optimal division pattern under different optimization procedures. Same as Fig. 8, except both evolutionary and functional objective functions are used, *F* ^*evol*^+*F* ^*func*^. Different parameters for the function *F* ^*func*^ are used, as indicated

Why does the observed division pattern have a maximum in the lower part of the crypt? As they move upward, cells in the crypt begin to differentiate. The cells near the top of the crypt are terminally differentiated, and cannot undergo further divisions. Experimentally, it is known that differentiated cells undergo apoptosis before they are removed from the luminal surface [63]. To account for this fact, we included a “penalty function” for proliferating near the top of the crypt (see Methods). For all parameter choices, this function (*F*^*func*^) penalizes the “top-high” over the “bottom-high” division patterns (see Fig. 4). Including this effect by considering the additive objective function, *F*^*evol*^+*F*^*func*^, we find (see Fig. 9) that for a large parameter ranges for function *F*^*func*^, the experimentally obtained division probability distribution function performs very well, or better, compared to other functional choices that we studied. Of course, the actual values of parameters *a* and *b* in the expression for *F*^*func*^ are unknown, and it is possible to find parameter combinations that change the “optimality” ordering of the different division probability distribution functions. Our goal here is to demonstrate that by adding functional considerations, the optimal probability distribution of divisions will shift from the top to lower parts of the crypt. Interestingly, we could not find a choice of parameters for the penalty function that makes the delta division distribution optimal.

To summarize, we note that if only considerations of double-hit mutant production are included, then the best strategy for a crypt is to have only top cells divide. In reality however, divisions and functionality of cells are often in a trade-off, and adding the proliferation ceiling will change the outcome of the optimization problem. Since terminally differentiated cells (situated at the top of the crypt) cannot functionally divide, the next best solution is to have intermediate (but not bottom) cells perform most of the divisions, which is consistent with the observations.

**Symmetric divisions delay two-hit mutant production, but the location of divisions is more important than divisions’ type**

In previous, non-spatial models, it was shown that symmetric stem cell divisions help delay the production of double-hit mutant compared to asymmetric stem cell divisions [19, 20, 22]. In the present model, the same trend is observed (see e.g. Fig. 5), but the magnitude of the effect is relatively small (see e.g. Fig. 6).

The most direct comparison between the non-spatial model of [20] and the present model is possible for the delta-distribution of divisions. This model for a two-row of cells corresponds exactly to the model of [20] with *S*=2 stem cells and no double-hit mutants produced in the differentiated compartment. The delta-distribution model in the cylindrical arrangement corresponds to a spatial (nearest neighbor) generalization of the model of [20]. In the latter paper, the difference between symmetric and asymmetric stem cell divisions was very significant. A mutant produced in the stem cell compartment will stay in the compartment indefinitely under the pure asymmetric divisions assumption. In contrast to that, purely-symmetric divisions allow for an opportunity for a mutant stem cell to be washed out (by a symmetric differentiation), thus leading to an overall decrease of the probability of double-hit mutant generation.

Building on this result, on the one hand, we have a very slow 2-hit mutant production by the symmetrically dividing stem cells, and on the other hand, a relatively fast 2-hit mutant production by asymmetrically dividing stem cells. Where does the spatial, cylindrical model with the delta division distribution fit in? To answer this question, we note that the process with purely symmetric divisions is equivalent to a simple Moran process if only the stem cells are considered (here, differentiations are effectively death events while symmetric proliferations are birth events). The cylindrical model with the delta distribution is then exactly a 1D, nearest neighbor spatial generalization of this Moran process. It was shown in [25, 26] that in such a spatial model, 2-hit mutants are produced *faster* than in a non-spatial Moran process. Therefore, the rate of 2-hit mutant production in the delta-distribution cylindrical model of the present paper will be somewhere between the two extreme results for the symmetric and asymmetric divisions described in [20]. In other words, the effect of symmetry is somewhat weakened in the delta-distribution model considered here.

Next, if we turn to the other division probability distributions, we will see that the effect of symmetry is weakened even further. For any division pattern, the only cell locations that can retain a mutant indefinitely under the asymmetric division model correspond to the largest *x* in the support of the function *p*(*x*). In other words, only the lowest (closest to the crypt bottom) dividing row of cells can produce a mutant that will not be pushed toward the top, because there are no other cells dividing upstream from that location. Any other location produces mutants that will eventually be moved upstream by the progeny of cells dividing below them, even in the purely asymmetric division model. This means that the arguments of [20] only apply to a fraction of cell divisions, and the fraction is small in “top-high” division patterns.

We conclude that even though symmetry of divisions plays a certain role in minimizing 2-hit mutant production, its effect is relatively small compared with the effect of spatial location studied in the present model.

**Additonal random cell death delays two-hit mutant generation, and minimizes the difference between the experimentally observed division pattern and the top high division pattern**

Apoptosis is also observed to occur persistently, though with low frequency, within healthy crypts [64]. If the loss of a cell in the crypt were to alleviate contact inhibition and result in a cell division, this effect can be incorporated into the probability of division *p*_*div*_(*x*). We specifically test the effects of apoptosis on cells at lower positions in the crypt on the main finding of the model. We compare these effects using parameters for the human colon crypt in Fig. 7. We test the previously investigated proliferation curves, as well as the empirical division probability function obtained from the positional BrdU label index in the human colon crypt from [44] (see Fig. 7a).

In Fig. 7b and c, we obtain the probability of 2-hit mutant generation $P_{2 hit}^{gen}(T)$ at least in one location as the function of the probability of symmetric division *σ*. In Fig. 7b, we do simulations as before, where cell deaths happen only at the top of the crypt. In Fig. 7c, we show the results of simulations for the modified model, where 10 % of the deaths happen uniformly randomly in the middle of the crypt. Comparing Fig. 7b and c, we notice that the difference between $P_{2 hit}^{gen}(T)$ for the top-high division pattern and the observed division pattern becomes smaller for random cell death. Random cell death forces cell division to happen in the middle of the crypt, rather than the top of the crypt. Thus, random cell death raises the probability of two-hit mutant generation for the top-high division pattern. However, in terms of delaying two-hit mutant generation, the top-high division pattern is still optimal. In general, the location of mutation’s generation depends on the division probability function (See Additional file 1: Figure S2). When the most common division location changes randomly, then the probability that a one-hit mutant is chosen to divide decreases. Therefore, random cell death delays the formation of the two-hit mutants due to changes in location of divisions.

## Discussion

In this work, we investigated different scenarios for cell division and their effects on two-hit mutant production in a spatial setting. As cells at the top of the colon crypt are washed away, in order to maintain homeostasis, some cells in the crypt must divide. These divisions might happen anywhere in the crypt, and then cells would migrate to fill out the empty spaces. In this model, we assumed the probability that a cell divides depends on its location. We also considered two different division patterns, symmetric and asymmetric. The symmetric pattern includes differentiation (daughter cells are placed upstream from the division location) and proliferation (daughter cells are placed at the same level as the dividing cell). In the asymmetric division pattern, one of daughter cells stays at the same location and the other one is placed upstream.

This simplified geometry of our model allows for the investigation of the effects of proliferation kinetics and (a) symmetry of division on the rate of mutation accumulation. We were able to derive analytical solutions for several quantities of interest such as the rate of two-hit mutant production, and compare to the numerical results. We found that in the context of two-hit mutant production in a linear process, the probability distribution of divisions as a function of their location has a bigger impact of double-hit mutant production compared with the divisions’ symmetry. In this context, higher probability of two-hit mutant generation corresponds to the higher probability of division at the bottom of the crypt.

**Comparison to prior work** Our model of the crypt is highly idealized to retain only the most pertinent features. There are more complex computational models in the literature, which include other effects, such as cell adhesion [34, 35]. We focus our discussion on the findings of the stochastic models for mutation accumulation since they are most comparable to our model. Our model implicitly assumes a differentiation hierarchy, as only cells at similar or less differentiated stages can replace dead cells at the top. Although we apply our model to both the small and large intestine (colon), there are some subtle differences between different crypt types. Among the differences are that cells at the top of a colon crypt die and are removed, whereas cells at the top of an intestinal crypt move up to a villus and are removed at the top of the villus. In this work, cell death is being modeled as it occurs in colon crypts. Crypts in the small intestine contain both stem and Paneth cells intermingled at the bottom of the crypt [65]. Colonic crypts do not contain Paneth cells. The contribution of Paneth cells to maintenance of intestinal crypt architecture through regulation of Wnt signalling has recently been computationally modeled [46]. We do not consider these different cell types in this model. We also do not consider the effect of microenvironment on the stem cells, and external chemical signalling from Paneth cells that may influence stem cell dynamics. Instead, we consider a generalized proliferation curve that captures differences between cells at different positions in the crypt. We do not consider fitness advantages of first hit mutation, but it can be incorporated by making the probability division functions depending on fitness.

Our simulations, as well as analytical reasoning presented in Additional file 1, suggest that in the context of minimizing the probability of a 2-hit mutant generation in a given number of cell division cycles, it is optimal to have most (but not all) cell divisions occur at the top of the crypt. Note that, for example, if only the cell at the bottom of the crypt divides (the delta division pattern), for the mutation rate chosen it is guaranteed that a mutation will arise after 10,000 divisions. Once the cell at the bottom of the crypt is mutated, it remains in the crypt indefinitely. As previously shown for the linear process architecture, all mutations arising in non-stem cells within the crypt column are eventually flushed out of the crypt; only mutations arising in the stem cell have the ability to remain in the crypt and reach fixation [39]. Hence, for a given number of cell division cycles, it is always advantageous to have divisions occur near the top of the crypt, so that if a mutation occurs, it is more likely to be flushed out.

Note that Zhao and Michor [23] previously found that most divisions should occur at the bottom of a crypt in order to maximize the time to cancer. There are however differences between the two model formulations that account for this discrepancy. The crucial difference is that [23] do not consider the probability of two-hit mutant production from wild type cells. Instead, they start with an APC ^+/−^ mutation at a given position and calculate the time to APC ^−/−^ mutation (that is, they consider the conditional probability provided the first mutation has already occurred). They find that, given a mutation has occurred, it is more likely to be eliminated with a curve that has more proliferation at the bottom of the crypt. Interestingly, the predictions of [23] agree with ours when they consider the probability of second mutation occurring from one-hit mutants: it is always higher when the one-hit mutant is placed at the bottom of the crypt. Our model agrees that to remove an *existing* mutant removal it is optimal to have most cell divisions occur at the bottom of the crypt (see Additional file 1: Figure S3).

**Why the “top-high” arrangement is optimal: an intuitive explanation.**

**Fig. 10:**
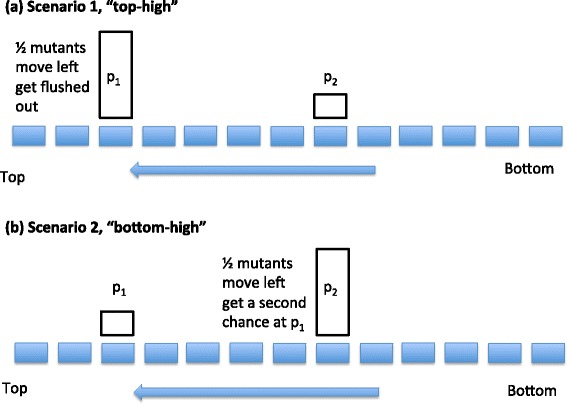
A comparison of the “*top-high*” and “*bottom-high*” scenarios in the case of asymmetric divisions. Here *p*_*div*_(*i*)=*p*_*i*_. **a** *p*_1_>*p*_2_>0. **b** 0<*p*_1_<*p*_2_

We showed above that “top-high” division probability distributions minimize the production of double-hut mutants. To understand this on an intuitive level, let us compare a “top-high” and a “bottom-high” division probably distribution functions, which consist of two peaks each: a high peak near the top (bottom) and a low peak near the bottom (top) of the crypt, respectively (Fig. 10).

For a top-high function, most mutants are generated near the top, remain there for a short time (during which they run a relatively high chance of a second hit because they are in the division hot spot), and they are pushed out relatively soon after that. Additionally there is a smaller chance of generating a mutant closer to the bottom. These mutants will be flushed out after a much longer period of time, and before that they will have to pass through the high-hit zone near the top of the crypt.

In the case of a bottom-high distribution, mutants are mostly generated near the bottom. They remain there for some time (again, running a relatively high chance of a second hit), and then they travel over some period of time toward the top of the crypt, during which time they pass over a spot where they have a smaller (but nonzero) chance of acquiring a second hit.

At the first glance, these two arrangements may appear equivalent. There is however an important difference. If the maximum division probability is located near the top of the crypt, the mutants produced at this spot can have two fates (see Fig. 10a): (1) either they remain at the spot and then they run a chance of further divisions (and thus may produce a two-hit mutant), or (2) they are flushed out to the top of the crypt without given a chance to divide. This happens because the division “hot-spot” is near the top of the crypt, and there are no other division spots downstream from that.

If the maximum division probability is located closer to the bottom of the crypt (see Fig. 10b), the mutants produced at this spot again can have two fates: (1) either they remain at the spot and then they run a chance of further divisions (and thus may produce a two-hit mutant), or (2) they move towards the top, but before they are flushed out they are given a second (smaller) chance to divide and produce two-hit mutants. This second change is what makes this configuration produce double-hit mutants faster, and thus makes the first configuration optimal. Analytical calculations supporting this argument are presented in the Additional file 1: Figure S7.

**Effects of proliferation ceiling.**

Our analysis shows that to minimize the probability of double-hit mutant production, cells should divide predominantly close to the top of the crypt. A possible selection pressure resulting from this trend is, however, counterbalanced by a requirement of a different nature, where cells’ functionality trades off with their proliferative potential. A proliferation ceiling based on an external Wnt and Bmp gradients is often imposed in computational models of the crypt [33, 35, 37, 46]. Interestingly, if no proliferation ceiling exists, i.e. BMP gradient is inhibited, the geometry of the crypt becomes perturbed, with new ectopic crypts appearing, and ultimately leads to the growth of juvenile polyps and to neoplasia [66]. Suggestive results are also observed in a spatial model of the crypt, where allowing unlimited proliferation near the top of the crypt results in splitting of the crypt through a fingering instability [67]. As our model does not focus on the structural integrity of the crypt, we set up an optimization problem where two selection forces were included: one coming from the need to minimize mutations, and the other from maximizing functionality (proliferation ceiling). The inclusion of the latter component resulted in the shift of division probability distribution away from the very top of the crypt toward its lower parts.

## Conclusions

Our overall conclusion is that it is advantageous for cells in the intestinal crypt to proliferate as close to the crypt’s top as possible, while still obeying the proliferation ceiling. Our work highlights the importance of considering both proliferation kinetics and the spatial organization of tissues when investigating the dynamics of cancer initiation.

## Reviewers’ comments

### Reviewer’s report 1

David Axelrod, Rutgers University (nominated by Marek Kimmel)

**Reviewer comments:** The authors provide a stochastic model of cell dynamics in colon crypts taking into account symmetric and asymmetric stem cell divisions, two? hit mutations, and spacial gradients of probability of cell division and cell death along the crypt axis. This is extension of previous modeling work, includes simulation results in comparison with published experimental observations, and reaches new conclusions. It contributes to the theme of this Special Issue by considering evolutionary and functional penalties of double-hit mutations. There is an ambiguity that needs to be resolved, whether colon crypts (page 4, line 26) or intestinal crypts (Title and Abstract) are being modeled. There are differences and there are similarities. Among the differences are that cells at the top of a colon crypt die and are removed, whereas cells at the top of an intestinal crypt move up to a villus and are removed at the top of the villus. The model seems to be of colon crypts, since cell death is assumed to always occur at the top of the crypt, x=0. Among the similarities are the organization of the three major cell types, stem cell divisions, and movement of cells up the crypt. Information about Wnt gradients along the crypt cited on page 5, and location of proliferating cells cited in the legend to Fig. 3 are correctly indicated as from murine intestinal crypts. Where information is obtained from mouse intestinal crypts, it is indicated, such as in legend to Fig. 3, or page 6, line 17; where information is obtained from human colon crypts, it is indicated, such as, page 6, lines 25–35. This is also done well in the section on Parameter estimates. Differences between intestinal and colon crypts for some cell types are discussed explicitly late in the text, on page 13. Perhaps to resolve the ambiguity in other places, about whether intestinal or colon crypts are being modeled, it should be stated early that the organization of cell types (stem cells, proliferating transient amplifying cells, and differentiated cells) and cell movement are similar in intestinal and colonic crypts, and cell death is being modeled as it is in colon crypts.

Authors’ response: *We have changed the title to “colon and intestinal crypts” to reflect that some of our simulations are parametrized for the colon, and some for the intestine. We added additional text to indicate the differences between the two model systems, and that our model is applicable to both structures*.

### Reviewer’s report 2

Yang Kuang, Arizona State University

**Reviewer comments:** This manuscript continues the timely and active discussion on the role of cell location and spatial gradients in the evolutionary dynamics of the intestinal crypt. It contains a good amount of background and modeling review of the related work. In many places the authors mentioned observed patterns of cell divisions in colonic crypts or the experimentally observed division pattern and the top high division pattern. These important observation motivated much of the work of this paper and other cited ones. For example, it prompted the authors to ask Why does the observed division pattern have a maximum in the lower part of the crypt? However, the authors did not elaborate these observed division patterns nor cite any references after those statements. I suggest the authors explicitly state these experimentally observed pattern of cell division and cite a few references. I would also suggest that the authors explicitly list their model key assumptions and provide some references to back them up, if possible.

Authors’ response: *We have made the model assumptions about crypt geometry, cell death, cell division and motility more clear in the section**Methods*. *We describe the experimentally observed division patterns and how they were measured in section Parameter estimations. References for the experimental curves in murine intestinal crypts (Buske 2011) and human colonic crypts (Potten, 1992) are provided in the text and Figures where those proliferation curves are shown. Other parameter estimates are given in Table**1*. *Model limitations are discussed further in the**Discussion*.

### Reviewer’s report 3

Anna Marciniak-Czochra, Heidelberg University

**Reviewer comments:** The paper is devoted to a numerical and analytical study of a stochastic model of cell differentiation in an intestinal crypt. The authors propose a new model accounting for a spatial (bottom-up) organisation of the differentiation process and for symmetric and asymmetric stem cell divisions. The aim is to understand how the two aspects influence a production of two-hit mutants. A particular attention is put to a role of different proliferation patterns (dependence of proliferation rates on the position along the crypt). It is shown that a higher probability of division at the bottom of the crypt, where the stem cells are located, leads to a higher rate of double-hit mutant production, while the optimal scenario (delaying the establishment of a mutant) takes place when most of the cell divisions happen at the top of the crypt. Taking into account the proliferation ceiling (related to a lack of functionality of the cells at the very top of the crypt) shifts the optimal high proliferation zone to the middle of the crypt. The spatial heterogeneity in respect to the proliferation rates appears to be more significant for the mutation accumulation than the heterogeneity in respect to the self-renewal properties (divisions’ symmetry). The study involves comprehensive numerical simulations supported by analytical calculations for several quantities of interest such as the rate of two-hit mutant production. It is discussed in the context of currently available data and existing models. I find this work timely and novel. It is a step towards better understanding of cancer evolution in stem cell initiated systems. I recommend its publication in Biology Direct. I do not see a need for any major revision.

Author response: *We thank the reviewer.*

## Abbreviations

Apc, adenomatous polyposis coli; BrdU, bromodeoxyuridine; TA, transit amplifying

## Additional file

Additional file 1 Supplementary Material. (PDF 642 kb)
