# Supplementary material for: The role of cell location and spatial gradients in the evolutionary dynamics of colon and intestinal crypts
Source: Biol Direct. 2016 Aug 23;11(1):42. doi: 10.1186/s13062-016-0141-6 (PMC4994304; doi:10.1186/s13062-016-0141-6)
Supplement: Supplementary file 1 — Supplementary Material. (PDF 642 kb) [file 13062_2016_141_MOESM1_ESM.pdf]

Supplementary material

Considering two-hit mutants at location $x = 0$

In the main text, our algorithm assumed that cells at the location $x = 0$ die at each updating time step. Hence, the calculated probability of two-hit mutant generation excluded the two-hit mutants that may have been generated at location $x = 0$. Here, we compute the amended probability of two-hit mutant generation including two-hit mutants at location $x = 0$. Figure S1b shows the result of this assumption comparing the probability of 2-hit mutant production with $P_{2hit}^{gen}(T)$ that excluded two-hit mutants at $x = 0$ (Figure S1a). In Figure S1b, for the top high division pattern become higher than $P_{2hit}^{gen}(T)$ in the previous model, but remains the optimal probability distribution function.

Analytical tools

In order to calculate the probability of double-hit mutant generation at each location, we first obtain the probability of 2-hit mutant production at the bottom of the crypt, i.e. the location $x = n - 1$.

Probability of 2-hit mutant production at the bottom of the crypt

Let p be the probability of division at the bottom of the crypt, i.e. $p = p_{div}(n - 1)$. We calculate the probability of two-hit mutant production for symmetric and asymmetric divisions patterns separately.

Asymmetric division

Since in the model for the asymmetric division, there is no difference between two cells in the location $x = n-1$, so we assume there is only one cell at this location. At each updating step, with probability p the cell at the location $n - 1$ will divide. With probability $\frac{u_1}{2}$, the offspring which is placed in the same location as its wild type mother cell is a 1-hit mutant . Moreover, the offspring replacing the divided 1-hit mutant mother cell is a two-hit mutant with probability $\frac{u_2}{2}$. Thus the probability of production of 2-hit mutant in the case of asymmetric division is obtained by the following system of ordinary differential equations (homogeneous state approximation):

$$\frac{\partial P_0(n-1,t)}{\partial t} = -0.5u_1 p P_0(n-1,t), \tag{1}$$

$$\frac{\partial P_1(n-1,t)}{\partial t} = 0.5p\left(u_1 P_0(n-1,t) - u_2 P_1(n-1,t)\right), \tag{2}$$

$$\frac{\partial P_2(n-1,t)}{\partial t} = 0.5u_2 p P_1(n-1,t), \tag{3}$$

$$P_0(n-1,0) = 1,\ P_1(n-1,0) \ = \ 0,\ P_2(n-1,0) \ = \ 0, \tag{4}$$

where $P_0(n-1,t),\ P_1(n-1,t),\ P_2(n-1,t)$ are respectively the probability of having wild type, one-hit mutants, and double-hit mutants at time t at the location $x = n - 1$. This system of equations implies the probabilities of 2-hit mutant and 1-hit mutant production at the bottom of the crypt are

1. if $u_1 \neq u_2$ then

$$P_0(n-1,t) = e^{-0.5ptu_1} \tag{5}$$

$$P_1(n-1,t) = \frac{u_1\left(e^{-0.5ptu_2} - e^{-0.5ptu_1}\right)}{u_1 - u_2} \tag{6}$$

$$P_2(n-1,t) = \frac{u_1(1-e^{-0.5ptu_2}) - u_2(1-e^{-0.5ptu_1})}{u_1 - u_2} \tag{7}$$

2. if $u_1 = u_2$ then

$$P_0(n-1,t) = e^{-0.5ptu_1} \tag{8}$$

$$P_1(n-1,t) = 0.5ptu_1 e^{-0.5ptu_1} \tag{9}$$

$$P_2(n-1,t) = 1 - e^{-0.5ptu_1}\left(1 + 0.5ptu_1\right). \tag{10}$$

Symmetric division

Here for the symmetric pattern, we obtain the transition matrix for the cells at the position $x = n-1$. At each location, there are two cells, so for the location $x = n-1$, there are 9 different states; $S_1 := \{0,0\}$, $S_2 := \{1,0\}$, $S_3 := \{0,1\}$, $S_4 := \{1,1\}$, $S_5 := \{2,0\}$, $S_6 := \{0,2\}$, $S_7 := \{2,2\}$, $S_8 := \{2,1\}$, $S_9 := \{1,2\}$, where 0 means no mutation, 1: 1-hit mutant, and 2: two-hit mutant. We calculate the transition matrix, the value at the row i and the column j corresponds to the probability of transforming from the state i to the state j.

$$T_s = \begin{pmatrix} 1-pu_1 & \frac{pu_1}{2} & \frac{pu_1}{2} & 0 & 0 & 0 & 0 & 0 & 0 \\ \frac{p(1-u_1)}{2} & p(\frac{u_1}{4}-1)+1 & \frac{pu_1}{4} & \frac{p(1-u_2)}{2} & 0 & 0 & 0 & \frac{pu_2}{4} & \frac{pu_2}{4} \\ \frac{p(1-u_1)}{2} & \frac{pu_1}{4} & p(\frac{u_1}{4}-1)+1 & \frac{p(1-u_2)}{2} & 0 & 0 & 0 & \frac{pu_2}{4} & \frac{pu_2}{4} \\ 0 & 0 & 0 & 1-pu_2 & 0 & 0 & 0 & \frac{pu_2}{2} & \frac{pu_2}{2} \\ \frac{p(1-u_1)}{2} & \frac{pu_1}{4} & \frac{pu_1}{4} & 0 & 1-p & 0 & \frac{p}{2} & 0 & 0 \\ \frac{p(1-u_1)}{2} & \frac{pu_1}{4} & \frac{pu_1}{4} & 0 & 0 & 1-p & \frac{p}{2} & 0 & 0 \\ 0 & 0 & 0 & 0 & 0 & 0 & 1 & 0 & 0 \\ 0 & 0 & 0 & \frac{p(1-u_2)}{2} & 0 & 0 & \frac{p}{2} & p(\frac{u_2}{4}-1)+1 & \frac{pu_2}{4} \\ 0 & 0 & 0 & \frac{p(1-u_2)}{2} & 0 & 0 & \frac{p}{2} & \frac{pu_2}{4} & p(\frac{u_2}{4}-1)+1 \end{pmatrix} \tag{11}$$

This transition matrix implies that the cell at the location $x = n-1$ is a two-hit mutant or 1-hit mutant at time t, with the following probabilities:

$$\vec{v} = (T_s')^t < 1,0,0,0,0,0,0,0,0 > \tag{12}$$

$$P_1(n-1,t) = v_2 + v_4 + v_9 \;=\; v_3 + v_4 + v_8 \tag{13}$$

$$P_2(n-1,t) = v_5 + v_7 + v_8 \;=\; v_6 + v_7 + v_9, \tag{14}$$

where v_i is the i-th element of $\vec{v}$. We numerically calculate v after t time steps.

Probability of two-hit mutation at each location

Recall that $x = 0$ corresponds to top of the crypt, and $x = n-1$ to the bottom. We can write a recursive equation to obtain the probability of 2-hit mutant production at each location x. Note that for both symmetric and asymmetric divisions' patterns, if a division occurs at each location $i < x$ then position of cells at the location x does not change. Furthermore, due to the spatial architecture of this model, both asymmetric and symmetric divisions result in two cells at position x and two cells position $x-1$. Also if the divisions happen at the location $i > x+1$, then cells at the location $x+1$ would move to the location x. The probability of having no mutations at position x at time t is:

$$\begin{aligned} P_0(x,t) &= \left(1 - \frac{u_1}{2}\right)(p_{div}(x+1)P_0(x+1,t-\Delta t) + p_{div}(x)P_0(x,t-\Delta t)) \\ &+ \sum_{i=1}^{x-1} p_{div}(i)P_0(x,t-\Delta t) + \sum_{i=x+2}^{n-1} p_{div}(i)P_0(x+1,t-\Delta t). \end{aligned} \tag{15}$$

Using $\sum_{i=1}^{x-1} p_{div(i)} = 1 - \sum_{i=x}^{n-1} p_{div(i)}$, we can rewrite the equation as

$$\begin{aligned} P_0(x,t) - P_0(x,t-\Delta t) &= P_0(x+1,t-\Delta t)\left((1-\frac{u_1}{2})p_{div}(x+1) + \sum_{i=x+2}^{n-1} p_{div}(i))\right) \\ &+ P_0(x,t-\Delta t)\left((1-\frac{u_1}{2})p_{div}(x) - \sum_{i=x}^{n-1} p_{div}(i)\right) \\ &= (P_0(x+1,t-\Delta t) - P_0(x,t-\Delta t)) \sum_{i=x+1}^{n-1} p_{div}(i) \\ &+ -\frac{u_1}{2}\left(p_{div}(x+1)P_0(x+1,t-\Delta t) + p_{div}(x)P_0(x,t-\Delta t)\right). \end{aligned}$$

Taking a Taylor expansion, and assuming $u_i(p_{div}(x+1)P_i(x+1) + p_{div(x)}P_i(x))/2 \approx u_i p_{div}(x)P_i(x,t)$ results in the following advection-reaction equation

$$\frac{\partial P_0(x,t)}{\partial t} = \sum_{i=x+1}^{n-1} p_{div}(i)\frac{\partial P_0}{\partial x} - u_1 p_{div}(x)P_0(x,t). \tag{16}$$

The probability of having one mutation at time x at time t is given by

$$\begin{aligned} P_1(x,t) &= p_{div}(x+1)\left(\frac{u_1}{2}P_0(x+1,t-\Delta t) + (1-\frac{u_2}{2})P_1(x+1,t-\Delta t)\right) \\ &+ p_{div}(x)\left(\frac{u_1}{2}P_0(x,t-\Delta t) + (1-\frac{u_2}{2})P_1(x,t-\Delta t)\right) \\ &+ \sum_{i=1}^{x-1} p_{div(i)}P_1(x,t-\Delta t) + \sum_{i=x+2}^{n-1} p_{div}(i)P_1(x+1,t-\Delta t). \end{aligned}$$

This can be rewritten as

$$
\begin{aligned}
P_1(x,t) - P_1(x,t-\Delta t) = {} & P_0(x+1,t-\Delta t)\left(\frac{u_1}{2}p_{div}(x+1)\right) + P_0(x,t-\Delta t)\left(\frac{u_1}{2}p_{div}(x)\right) \\
& + P_1(x+1,t-\Delta t)\left((1-\frac{u_2}{2})p_{div}(x+1) + \sum_{i=x+2}^{n-1} p_{div}(i)\right) \\
& + P_1(x,t-\Delta t)\left((1-\frac{u_2}{2})p_{div}(x) - \sum_{i=x}^{n-1} p_{div}(i)\right).
\end{aligned}
$$

Again, assuming $p_{div}(x) \approx p_{div}(x+1)$, and keeping only the highest order terms in the Taylor expansion, result in the following advection-reaction equation

$$
\frac{\partial P_1(x,t)}{\partial t} = \sum_{i=x+1}^{n-1} p_{div}(i)\frac{\partial P_1}{\partial x} + u_1 p_{div}(x)P_0(x,t) - u_2 p_{div}(x)P_1(x,t). \tag{17}
$$

Similarly, we obtain an equation for $P_2(x,t)$

$$
\frac{\partial P_2(x,t)}{\partial t} = \sum_{i=x+1}^{n-1} p_{div}(i)\frac{\partial P_2}{\partial x} + u_2 p_{div}(x)P_1(x,t). \tag{18}
$$

Equations (17), (18), (19) are linear advection reactions, which could be solved analytically by method of characteristics. The advection velocity at x is dependent on the division gradient probability for the cells below x, so cells that are near the top of the crypt are removed faster. The boundary conditions for the equations are given by the ODEs governing the probability of mutant production at the bottom of the crypt (8) and initial condition is given by (5).

Note that we can numerically obtain the probability of 2-hit mutant production by solving equations (17), (18), (19) in steady state:

$$
\frac{\partial P_0}{\partial x} = \frac{u_1 p_{div}(x)P_0(x,t)}{\sum_{i=x+1}^{n-1} p_{div}(i)}, \tag{19}
$$

$$
\frac{\partial P_1}{\partial x} = p_{div}(x)\frac{u_2 P_1(x,t) - u_1 P_0(x,t)}{\sum_{i=x+1}^{n-1} p_{div}(i)}, \tag{20}
$$

$$
\frac{\partial P_2}{\partial x} = -\frac{u_2 p_{div}(x)P_1(x,t)}{\sum_{i=x+1}^{n-1} p_{div}(i)}. \tag{21}
$$

Equation (22) implies that for large t if $u_2 p_{div}(x) \ll 1$, $\frac{\partial P_2}{\partial x} \approx 0$. Hence, when the number of cells n is large and mutation rates are small $P_2(i+1,t) \approx P_2(i,t)$ for all $1 < i < n$.

Time for mutant removal is minimized when most cell divisions occur at the bottom

We also computed the removal velocity $\sum_{i=x+1}^{n-1} p_{div}(i)$ of cells at position x per cell division in Figure S3. In accord with [20] we also find that the velocity at which cells are removed from the crypt increases for cells higher in the crypt. Note, that with exception of the delta pattern, the fastest velocity occurs for the experimentally observed division pattern, and the cells at position x are removed faster when divisions are more likely in the lower part of the crypt. From the velocity, we can calculate the estimated time $\tau_i = i/p_{div}(i)$ needed to remove a mutant at position i (Figure S3c), which again is minimized when all divisions occur at the bottom of the crypt. Note that we can also calculate the expected time to remove a mutant that arises anywhere in the crypt $\int_i p_{div}(i)\tau_i$. The most optimal design for eliminating already occurring mutations is once again delta, where all divisions take place at the lowest position, with experimentally observed division pattern also performing very well. However, as we showed, delta is the worst division pattern for accumulating mutations, with a two-hit mutant guaranteed to arise when the probability of getting a two-hit mutant for the experimentally observed divisions is less than half (see Figure 9).

Model System with Two Possible Division Location

Suppose that the crypt has length $n = 4$, where $x = 0$ is the top of the crypt and $x = 3$ is the bottom, as before. Further assume that the division probability distribution only has two nonzero values, $p(1) = p_1$ and $p(2) = p_2$, such that $p_1 + p_2 = 1$ (see Figure 10). Here $p_{div}(i) = p_i$. We assume strictly asymmetric divisions and therefore only considering one row of cells is sufficient. What is the optimal pair (p_1, p_2) to minimize double-hit mutant production?

Since cells can only divide at locations $x = 1$ and $x = 2$, the system can be described as a Markov walk over the following 5 states: $(0, 0)$, $(1, 0)$, $(0, 1)$, $(1, 1)$, and E, where the first four states are scribed as the pairs of zeros and ones, where zero corresponds to the wild-type cells and ones to one-hit mutant cells, at locations $(1, 2)$. The last state corresponds to the existence of a double-hit mutant. The 5×5 transition matrix is given by

$$M = \begin{pmatrix} p_1\left(1-\frac{u_1}{2}\right)+p_2(1-u_1) & \frac{p_1u_1}{2}+\frac{p_2u_1}{2} & \frac{p_2u_1}{2} & 0 & 0 \\ p_2(1-u_1) & p_1(1-u_2)+\frac{p_2u_1}{2} & \frac{p_2u_1}{2} & 0 & p_1u_2 \\ 0 & 0 & p_1\left(1-\frac{u_1}{2}\right) & \frac{p_1u_1}{2}+p_2(1-u_2) & p_2u_2 \\ 0 & 0 & 0 & 1-u_2 & u_2 \\ 0 & 0 & 0 & 0 & 1 \end{pmatrix}$$

The initial state is given by a row-vector $X_0 = (1, 0, 0, 0, 0)$, where each entry is the probability to find the system in each of the 5 states. The probabilities after t divisions are given by $X_0 M^t$.

We can also consider a 4-state system by setting $u_2 = 0$ (the same states as before except E). We

have

$$M_0 = \begin{pmatrix} p_1\left(1-\frac{u_1}{2}\right)+p_2(1-u_1) & \frac{p_1u_1}{2}+\frac{p_2u_1}{2} & \frac{p_2u_1}{2} & 0 \\ p_2(1-u_1) & p_1+\frac{p_2u_1}{2} & \frac{p_2u_1}{2} & 0 \\ 0 & 0 & p_1\left(1-\frac{u_1}{2}\right) & \frac{p_1u_1}{2}+p_2 \\ 0 & 0 & 0 & 1 \end{pmatrix}$$

This matrix is the key to understanding the difference between the top-high and bottom high scenarios. Large values of p_2 create relatively large entries above the main diagonal, creating and retaining one-hit mutants.

Starting from $X_0 = (1, 0, 0, 0)$, the probability to produce a double-hit mutant at step t (given that it hasn't been produced so far) is given by

$$H_t = X_0 M_0^t S u_2, \tag{22}$$

where vector S is defined as,

$$S = (0, p_1, p_2, 1)^T$$

It is possible to show that the first term in the Taylor expansion of H_t in terms of small u_1 is given by

$$H_t = X_0 M^t \approx \frac{1}{2}\left(t(1-p_1) + \frac{p_1^2(1-p_1^t)}{1-p_1}\right) u_1 u_2,$$

where we used $p_2 = 1 - p_1$. Note that functions H_n satisfy $H_t = t/2$ both for $p_1 = 0$ and $p_1 = 0$, and for intermediate values of p_1 they have a "dip"-shape with one minimum. The minimum corresponds to $p_1 = 1/2$ for $n = 1$, and it corresponds to increasing values of $p_1 > 1/2$ as n increases. For large values of n and $0 < p_1 < 1$ we have

$$H_\infty = \frac{1}{2}\left(t(1-p_1) + \frac{p_1^2}{1-p_1}\right) u_1 u_2,$$

and the minimum of this function is located at

$$p_1 \approx 1 - \frac{1}{\sqrt{t}}.$$

We conclude the following. In the presence of at most two dividing locations next to each other,

- The maximum of the hazard function corresponds to $p_1 = 0$ or $p_1 = 1$. These are both delta-distributions of division probabilities. They are the worst design. Interestingly ,it does not matter where the delta is located. This result generalizes to any number of cells in the system ($n > 3$).
- If $0 < p_1 < 1$, the optimal solution will have $p_1 > 1/2$, that is, a "top-high" distribution leads to a lower rate of mutant production. For low rates u_2, it takes a long time to produce a mutant, so hazard functions for large t become important. In this limit, $p_1 = 1/\sqrt{t}$, that is, is it close to one (but is not quite one).

Model System with Three Possible Division Location

If we have three locations where cells can potentially divide ($x = 1, 2, 3$ with corresponding probabilities p_1, p_2, p_3, $\sum_i p_1 = 1$), we can have eight states, $(0,0,0)$, $(1,0,0)$,$(0,1,0)$, $(0,0,1)$, $(1,1,0)$, $(0,1,1)$, $(1,0,1)$,$(1,1,1)$ with 0,1, and 2 one-hit mutants and the state E with a double-hit mutant. In the $u_2 = 0$ case, the transition matrix is given by $M_0 =$

$$M = \begin{pmatrix} p_1\left(1-\frac{u_1}{2}\right)+(1-u_1)(p_2+p_3) & \frac{p_1u_1}{2}+\frac{p_2u_1}{2} & \frac{p_2u_1}{2}+\frac{p_3u_1}{2} & \frac{p_3u_1}{2} & 0 & 0 & 0 & 0 \\ p_2(1-u_1)+p_3(1-u_1) & p_1+\frac{p_2u_1}{2} & \frac{p_2u_1}{2}+\frac{p_3u_1}{2} & \frac{p_3u_1}{2} & 0 & 0 & 0 & 0 \\ 0 & p_3(1-u_1) & p_1\left(1-\frac{u_1}{2}\right) & 0 & \frac{p_1u_1}{2}+p_2+\frac{p_3u_1}{2} & 0 & \frac{p_3u_1}{2} & 0 \\ 0 & 0 & 0 & p_1\left(1-\frac{u_1}{2}\right)+p_2(1-u_1) & 0 & \frac{p_2u_1}{2}+p_3 & \frac{p_1u_1}{2}+\frac{p_2u_1}{2} & 0 \\ 0 & p_3(1-u_1) & 0 & 0 & p_1+p_2+\frac{p_3u_1}{2} & 0 & \frac{p_3u_1}{2} & 0 \\ 0 & 0 & 0 & 0 & 0 & p_1\left(1-\frac{u_1}{2}\right) & 0 & \frac{p_1u_1}{2}+p_2+p_3 \\ 0 & 0 & 0 & p_2(1-u_1) & 0 & \frac{p_2u_1}{2}+p_3 & p_1+\frac{p_2u_1}{2} & 0 \\ 0 & 0 & 0 & 0 & 0 & 0 & 0 & 1 \end{pmatrix}$$

In formula (23) we used

$$S = (0, p_1, p_2, p_3, p_1 + p_2, p_2 + p_3, p_1 + p_3, 1)^T.$$

We can derive an analogous analytical expression for the 1st term in the Taylor expansion of H_t in terms of small u_1:

$$\begin{aligned} a_1 &= p_1^t(3p_1 + 2p_2) - 2p_1(p_1 + p_2)^t + (p_1 + p_2)^{t+1} + t(-p_1 - p_2 + 1) - p_1, \\ a_2 &= (3p_2 - 1)p_1^{t+1} + p_1^{t+2} + 2(p_2 - 1)p_2p_1^t - p_1p_2\left(-(p_1 + p_2)^t + 3p_2 - 3\right) \\ &\quad + p_2\left(p_2 - (p_1 + p_2)^n\right) - p_1^3 + p_1^2(1 - 4p_2), \\ H_t &\approx \frac{u_1u_2}{2}\left(a_1 + \frac{a_2}{(1 - p_1)(1 - p_1 - p_2)}\right). \end{aligned} \tag{23}$$

where we used $p_3 = 1 - p_1 - p_2$. An example of this function is presented in figure S4, where the quantity H_t with $t = 10$ is plotted as a function of p_1 and p_2. We can see that it has a minimum at the interior of the simplex, relatively close to the $p_1 = 1$ corner.

It is interesting to see how this landscape changes with t. As expected, this function is equal to $t/2$ for $p_1 = 1$, $p_2 = 1$, or $p_3 = 1$. These are points of the maximum of this function. For $t = 1$, the minimum corresponds to $p_1 = p_3 = 1/2$. Starting from $t = 2$, the minimum of his function occurs in the interim of the simplex $p_1 + p_2 + p_3$ and depends on t. It is shown on figure S5, where it traces a curve on the simplex as t increases. For large values of t, we have $p_1 > p_2 > p_3$, and $\lim_{t\to\infty} p_2(t) = \lim_{t\to\infty} p_3(t) = 0$.

Another way to investigate the generation of double-hit mutants is to consider the transition matrix with a nonzero u_2,

$$M = \begin{pmatrix} p_1\left(1-\frac{u_1}{2}\right)+(1-u_1)(p_2+p_3) & \frac{p_1u_1}{2}+\frac{p_2u_1}{2} & \frac{p_2u_1}{2}+\frac{p_3u_1}{2} & \frac{p_3u_1}{2} & 0 & 0 & 0 & 0 & 0 \\ p_2(1-u_1)+p_3(1-u_1) & p_1(1-u_2)+\frac{p_2u_1}{2} & \frac{p_2u_1}{2}+\frac{p_3u_1}{2} & \frac{p_3u_1}{2} & 0 & 0 & 0 & 0 & p_1u_2 \\ 0 & p_3(1-u_1) & p_1\left(1-\frac{u_1}{2}\right) & 0 & \frac{p_1u_1}{2}+p_2(1-u_2)+\frac{p_3u_1}{2} & 0 & \frac{p_3u_1}{2} & 0 & p_2u_2 \\ 0 & 0 & 0 & p_1\left(1-\frac{u_1}{2}\right)+p_2(1-u_1) & 0 & \frac{p_2u_1}{2}+p_3(1-u_2) & \frac{p_1u_1}{2}+\frac{p_2u_1}{2} & 0 & p_3u_2 \\ 0 & p_3(1-u_1) & 0 & 0 & p_1(1-u_2)+p_2+\frac{p_3u_1}{2} & 0 & \frac{p_3u_1}{2} & 0 & (p_1+p_2)u_2 \\ 0 & 0 & 0 & 0 & 0 & p_1\left(1-\frac{u_1}{2}\right) & 0 & \frac{p_1u_1}{2}+(p_2+p_3)(1-u_2) & (p_2+p_3)u_2 \\ 0 & 0 & 0 & p_2(1-u_1) & 0 & \frac{p_2u_1}{2}+p_3(1-u_2) & p_1(1-u_2)+\frac{p_2u_1}{2} & 0 & (p_1+p_3)u_2 \\ 0 & 0 & 0 & 0 & 0 & 0 & 0 & 1-u_2 & u_2 \\ 0 & 0 & 0 & 0 & 0 & 0 & 0 & 0 & 1 \end{pmatrix}$$

This is a 9×9 matrix among the 8 states with 0,1, and 2 one-hit mutants and the state E with a double-hit mutant. We can calculate the probability to be in state E after t updates, which is given by $\{X_0M_0^t\}_9$,

the 9th component of the vector $X_0 M_0^t$. As the hazard function, this quantity reaches its maximum in the corners of the simplex, that is, for $p_1 = 1$, or $p_2 = 1$, or $p_3 = 1$. A simple expression can be derived for this quantity's leading term in the u-Taylor expansion, in the case where $p_3 = 0$ (i.e. $p_2 = 1 - p_1$):

$$\{X_0 M_0^t\}_9 \approx \frac{u_1 u_2}{4} \left(t(t-1)(1-p_1) + \frac{2p_1^2[t(1-p_1) + p_1^t - 1]}{(1-p_1)^2} \right). \qquad (24)$$

For large values of t, this quantity reaches its minimum at

$$p_1 \approx 1 - \sqrt{\frac{2}{t}}.$$

The maximum is given by

$$\{X_0 M_0^t\}_9 \approx \frac{u_1 u_2}{4} t(t-1),$$

and corresponds to $p_1 = 0$ or $p_1 = 1$. This shows the limit of applicability of the Taylor approximation: we must have

$$t \ll \frac{2}{\sqrt{u_1 u_2}}.$$

For large values of t (but within t his limit), we have a very simple, linear in p_1, formula:

$$\{X_0 M_0^t\}_9 \approx \frac{u_1 u_2}{4} t^2 (1-p_1), \quad 1 - p_1 \gg \sqrt{\frac{2}{t}}.$$

Figure S6 plots the comparison of the exact expression $\{X_0 M_0^t\}_9$ (dots) with approximation (25) (lines). Figure S7 plots the comparison of the approximation (25), exact expression $\{X_0 M_0^t\}_9$ and numerical simulations of the spatial Moran model. One can see that the approximation becomes better for smaller values of the mutation rate.

Conclusions

Our findings can be summarized as follows.

- As before, the maximum production of double-hit mutants corresponds to $p_1 = 1$, or $p_2 = 1$, or $p_3 = 1$ (the corners of the simplex, the delta-distributions, regardless of their location).
- The optimal distribution is characterized by $p_1 > p_2 > p_3 > 0$ (top-heavy).
- The optimal distribution must have a nonzero probability at all three locations ($x = 1, 2, 3$). Restricting the support of the distribution function increases the associated rate of double-hit mutant production.

Supplemental Figures

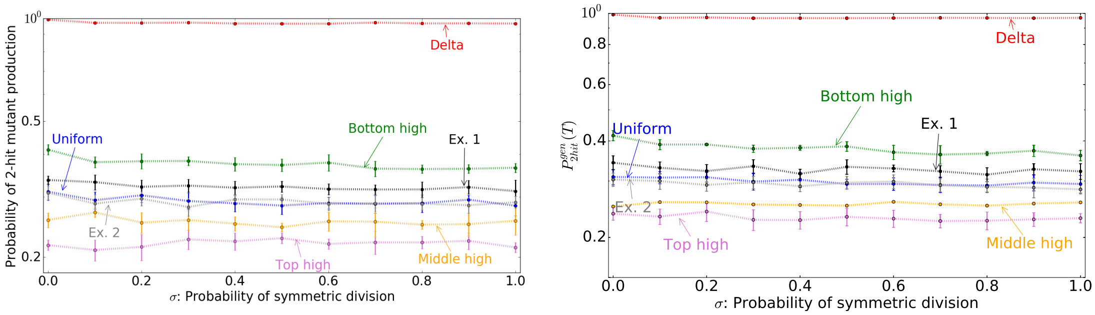

(a) Not considering 2-hits at $x = 0$

(b) Considering 2-hits at $x = 0$

Figure S1. Probability of two-hit mutant generation at the top of the crypt. The probability of 2-hit mutant generation $P^{gen}_{2hit}(T)$ is plotted for different probabilities of symmetric division, σ, for different division probability distribution functions. In this figure, we model entire mice colon crypt (i.e. 6 two-rows). We connect the results of simulations by dotted lines for each division probability function. The parameters are $T = 2000,\ u_1 = 0.005,\ u_2 = 0.005,\ n = 26$. The total number of cells in this simulation is 312. The lines Exp. 1 and Exp. 2 correspond to the division probability function that we obtained from experimental data in [33].

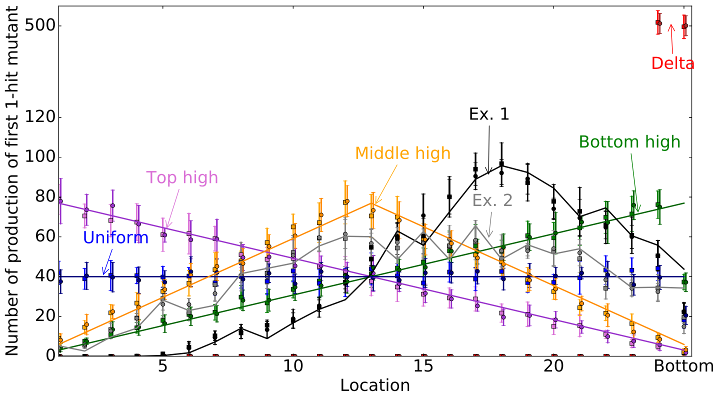

Figure S2. Histogram of first one hit mutant production at each location in the crypt. The number of one-hit mutants at each location in the crypt after a fixed time is proportional to the probability division function at that location $p_{div}(x)$. Here the total number of runs=1000 and solid lines shown are $1000 \cdot p_{div}(x)$. Parameters used are $u_1 = 0.001$, $u_2 = 0.005$, $t = 20,000$. Squares are symmetric divisions only ($\sigma = 1$), and circles are asymmetric ($\sigma = 0$).

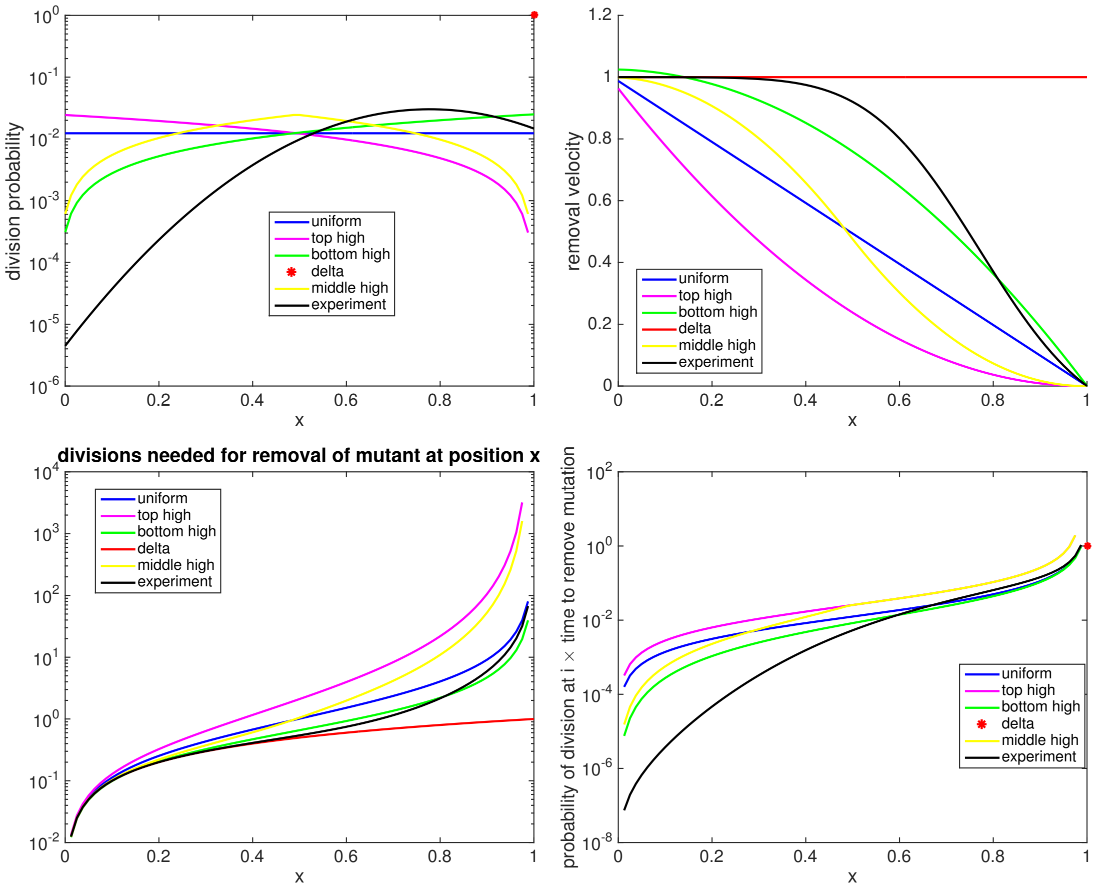

Figure S3. Removal of a mutant at position x. (a) Probability division functions considered. $x = 0$ corresponds to the top of the crypt, and $x = 1$ corresponds to bottom of the crypt. (b) Velocity $\sum_{i=x+1}^{n-1} p_{div}(i)$ at which cells are advected upwards. (c) The number of divisions needed to sweep away cell at x. (d) The probability-weighted value of time to remove a cell at position x.

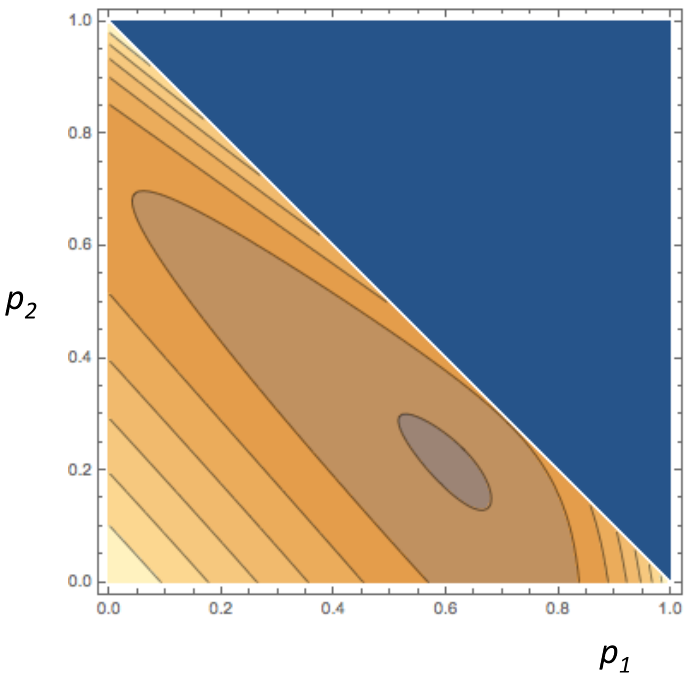

Figure S4. A heat-plot of the function H_t with three possible division locations equation with $t = 10$, as a function of p_1 and p_2 (with $p_3 = 1 - p_1 - p_2$). Lighter colors correspond to higher values.

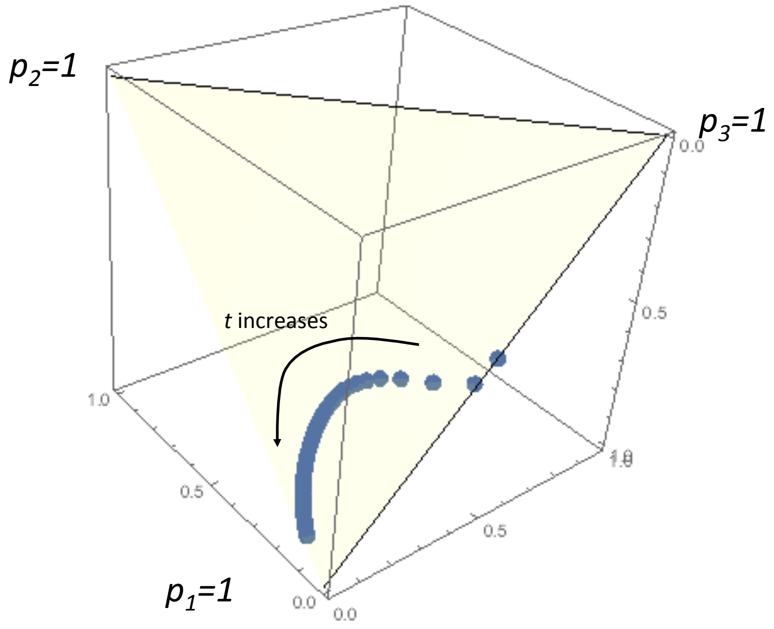

Figure S5. The locations of the point, $(p_1(t), p_2(t), p_3(t))$, that minimizes the function H_t. The axes are p_1, p_2, p_3, and the triangle represents the simplex $p_1 + p_2 + p_3 = 1$.

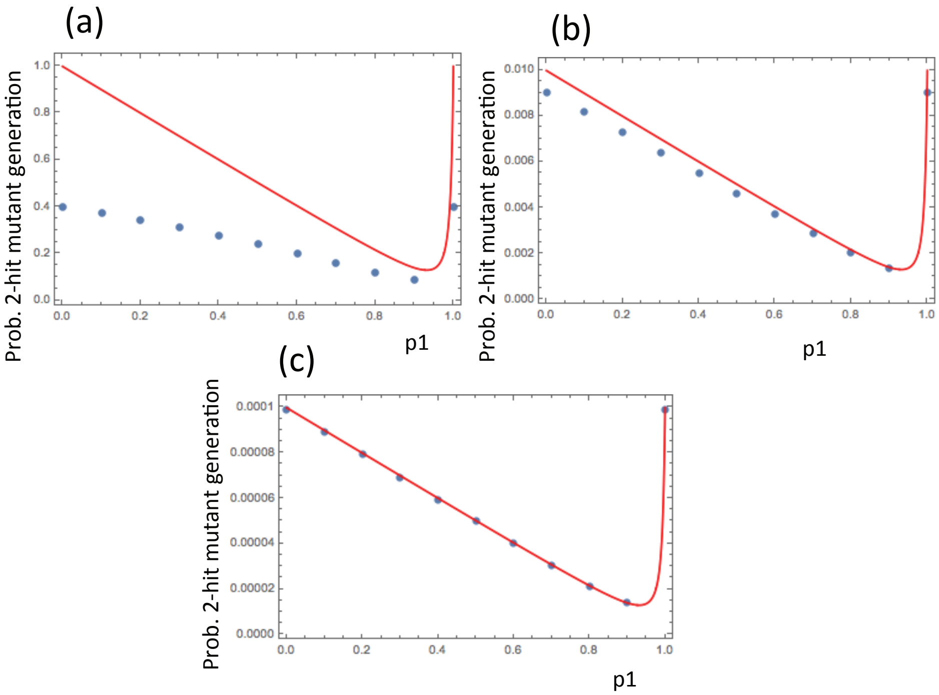

Figure S6. Analytical solution for probability of two-hit mutant with three possible division locations. The probability of two-hit mutant generation, after $t = 400$, plotted as a function of p_1, with $p_2 = 1 - p_1$ and $p_3 = 0$. The quantity $\{X_0 M_0^t\}_9$ (dots) is compared with approximation (25) (lines). (a) $u_1 = u_2 = 5 \cdot 10^{-3}$, (b) $u_1 = u_2 = 5 \cdot 10^{-4}$, (c) $u_1 = u_2 = 5 \cdot 10^{-5}$.

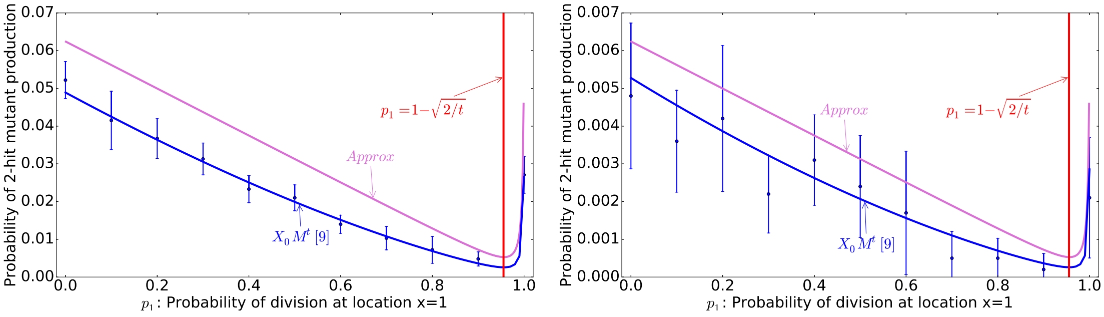

(a) **(b)**

Figure S7. Comparison of analytical and numerical solutions for probability of two-hit mutant with three possible division locations. The probability of two-hit mutant generation $P_{2hit}^{gen}(T)$ after $t = 1000$, plotted as a function of p_1, with $p_2 = 1 - p_1$ and $p_3 = 0$ for (a) $u_1 = u_2 = 5.\ 10^{-4}$ and (b) $u_1 = 5.\ 10^{-5}$, $u_2 = 5.\ 10^{-4}$. The results of numerical simulations (dots) and the quantity $X_0 M^t[9]$ (blue lines) are compered with the approximation formula (orchid line)
